# Dispersal patterns of oribatid mites across habitats and seasons

**DOI:** 10.1007/s10493-022-00686-y

**Published:** 2022-01-17

**Authors:** Peter Hans Cordes, Mark Maraun, Ina Schaefer

**Affiliations:** JFB Institute of Zoology and Anthropology, University of Göttingen, Untere Karspüle 2, 37073 Göttingen, Germany

**Keywords:** Phoresy, Walking, Dispersal mode, Season, Malaise traps, Active dispersal

## Abstract

Oribatid mites are tiny arthropods that are common in all soils of the world; however, they also occur in microhabitats above the soil such as lichens, mosses, on the bark of trees and in suspended soils. For understanding oribatid mite community structure, it is important to know whether they are dispersal limited. The aim of this study was to investigate the importance of oribatid mite dispersal using Malaise traps to exclude sole passive wind-dispersal. Oribatid mite communities were collected over a 3-year period from five habitat types (coniferous forests, deciduous forests, mixed forests, meadows, bog/heathlands sites) and three seasons (spring, summer, autumn) in Sweden. Mites entered traps either by walking or by phoresy, i.e., by being attached to flying insects. We hypothesized (1) that oribatid mite communities in the traps differ between habitats, indicating habitat-limited dispersal, and (2) that oribatid mite communities differ among seasons suggesting that dispersal varies due to changing environmental conditions such as moisture or resource availability. The majority of the collected species were not typically soil-living species but rather from habitats such as trees, lichens and mosses (e.g., *Carabodes labyrinthicus, Cymbaeremaeus cymba, Diapterobates humeralis* and *Phauloppia lucorum*) indicating that walking into the traps or entering them via phoresy are of greater importance for aboveground than for soil-living species. Overall, oribatid mite communities collected in the traps likely originated from the surrounding local habitat suggesting that long distance dispersal of oribatid mites is scarce. Significant differences among seasons indicate higher dispersal during warm and dry periods of the year. Notably, 16 species of oribatid mites collected in our study were sampled for the first time in Sweden. This study also demonstrates that Malaise traps are a meaningful tool to investigate spatial and temporal patterns of oribatid mite communities.

## Introduction

Dispersal is the movement of an organism away from its local area to another patch and is important for survival and reproduction of individuals, for community structure and dynamics of populations as well as for the distribution and evolution of species (Nathan 2001). Active dispersal via walking, jumping or flying allows targeting the destination site, but is less effective for very small animals, such as microarthropods, which can only cover a distance of a few centimeters to meters (Jenkins et al. 2007). In contrast, passive dispersal means that an organism is transported over large distances by wind (air-currents) or water bodies, but implies that the organism has no control of its landing site. Passive dispersal by wind is common for plant seeds, but also for wingless arthropods such as immature Lepidoptera, mites and spiders (Washburn and Washburn 1984), that may travel a few hundred meters to tens or even hundreds of kilometers by wind (Reynolds et al. 2007). Active and passive dispersal may also co-occur if animals like mites and spiders actively take postures or seek out exposed sites, which makes them carried away easily by wind (Washburn and Washburn 1984; Monteiro et al. 2018). Another interaction of active and passive dispersal is phoresy in which an animal (the phoront) actively attaches itself onto a host animal for the purpose of dispersal, enabling it to migrate to new habitats (White et al. 2017). This form of dispersal also reduces the uncertainty of destination sites compared to passive wind dispersal, because the host organism likely re-visits similar microhabitats at which the phoront may release itself.

Oribatid mites (Acari, Oribatida) are small, wingless arthropods with a body size ranging between 150 µm and 2 mm (Weigmann 2006). They typically inhabit the soil and litter layers, and active dispersal rates when walking on or in the soil have been estimated for various species to range between 0.6–29 cm per week (Ojala and Huhta 2001) and 0.3–2.9 cm per day (Lehmitz et al. 2012). However, many species are known to occur on trees where they occupy various microhabitats, such as bark, epiphytes such as lichen and mosses, twigs, suspended soils or phytotelmata (Behan-Pelletier and Walter 2000), and account for 34–88% of the arthropod fauna in canopies (Walter and O’Dowd 1995; Behan-Pelletier and Walter 2000; Lindo and Winchester 2006). Arboreal oribatid mite communities are distinct from those living in soil and litter (Behan-Pelletier and Walter 2000; Karasawa et al. 2005; Lindo and Winchester 2006; Lindo 2010) and show a significant species turnover between microhabitats and even between tree species within one forest (Walter and O’Dowd 1995; Winchester et al. 2008; Bolger et al. 2014; Hidasi-Neto et al. 2018), indicating that arboreal microhabitats are rather islands on a tree than a continuous habitat. Additionally, microclimatic fluctuations in, e.g., air-moisture and temperature, are more pronounced in arboreal microhabitats than below the ground (Wunderle 1992; Prinzing 2005), suggesting that tree-living oribatid mite species likely underlie high pressure to disperse between these ‘islands’ to have access to food and shelter (Bailey et al. 2018). A metacommunity experiment investigating recolonization of moss patches in fragmented habitats showed that oribatid mites rely strongly on active ground-based movement and therefore can be severely dispersal limited (Åström and Bengtsson 2011). However, long-distance transport by wind is common among oribatid mites and correlates positively with abiotic conditions such as strong wind speed, temperature or aridity, and is particularly common in individuals with a body size between 300 and 500 μm (Lehmitz et al. 2012). Arboreal oribatid mites disperse passively by wind or being attached to dispersal vectors such as falling leaf litter, branch tips and twigs that remain in the canopy (Karasawa et al. 2005; Lindo 2010). Studies that used Malaise and window traps in canopies concluded that random walking was an important mode of active dispersal (Behan-Pelletier and Winchester 1998; Karasawa et al. 2005). Direct observations of microarthropods on tree trunks concluded that tree-living oribatid mites move actively between microhabitats at small scales up to a few centimeters and decimeters, due to compensatory redistribution as response to microclimatic fluctuations (Prinzing 2005). Further, oribatid mite communities in tree canopies differed among vertical strata, indicating a complex structure of microhabitat use within canopy layers (Karasawa et al. 2005; Prinzing 2005; Lindo 2010). Among studies of tree-living oribatid mite communities, patterns of seasonal fluctuations were inconsistent (Walter and O’Dowd 1995; Behan-Pelletier and Winchester 1998; Yoshida and Hiji 2005; Lindo 2010). In temperate deciduous forests, temporal variation in chemistry and nutritional quality of arboreal resources have been suggested as potential factors driving seasonal arthropod variation in temperate deciduous forests (Valencia-Cuevas and Tovar-Sánchez 2015).

Phoresy as dispersal mode within canopies was considered to be of minor importance for most arboreal oribatid mites, because only few species in Malaise traps in canopies showed specific modifications for this dispersal mode (Behan-Pelletier and Winchester 1998). Phoretic associations between insects and oribatid mites likely are ancient (Robin et al. 2016). They appear to be not species-specific (Norton 1980); however, associations between bark beetles and oribatid mite species can be host-specific at least on a local scale (Knee et al. 2013; Knee 2017). Oribatid mites use a variety of host species, including Coleoptera, Dictyoptera, Diptera (Norton 1980), Hemiptera (Waleckx et al. 2018), and ground-living harvestmen (Opiliones) (Townsend et al. 2008), but some oribatid mite species also use birds (Krivolutsky and Lebedeva 2002, 2004a,b; Krivolutsky et al. 2004; Lebedeva 2012) and mammals (Miko and Stanko 1991) as hosts. The lack of special adaptations for attachment in oribatid mites also supports the loose phoretic relationship with insects. However, exceptions are the tropical genus *Mesoplophora* which has a tooth-like tubercle on each genital plate that aid to lock an insect’s hair that is clasped between the rostrum of the aspis and the anterior portion of the genital plates (Norton 1980), and a few species within the family Scheloribatidae that use a strong hook-like claw for clasping onto insect hairs (Schäffer and Koblmüller 2020; Ermilov and OConnor 2020).

In this study, we investigated the general importance of dispersal by walking actively or by phoresy for oribatid mites, by comparing communities from Malaise traps of different habitats. Oribatid mites cannot enter Malaise traps by passively floating in air-currents. They either walked from the tent or the ground into the collection vials, or detached from an insect host to which they actively attached in their local patch prior to transport, i.e., by phoresy (Karlsson et al. 2020). Traps were collected from 2003 to 2006 by the Swedish Malaise Trap Project (SMTP) across Sweden. The study design therefore provided a general picture of oribatid mite species that disperse by walking or phoresy because it covered a wide geographic range, a long sampling period and several habitats.

We analyzed whether (1) oribatid mite communities differ among habitats (coniferous, deciduous and mixed forests, bog/heath, meadows), indicating a limited ability of oribatid mites to disperse over large distances. Furthermore, we tested whether (2) oribatid mite communities differ between three seasons (spring, summer and autumn), suggesting that seasonal variation of microhabitat quality may induce oribatid mite dispersal. If oribatid mite communities do not differ among seasons, this would indicate stable populations throughout the year.

## Materials and methods

### Sampling and species determination and categorization

**Fig. 1:**
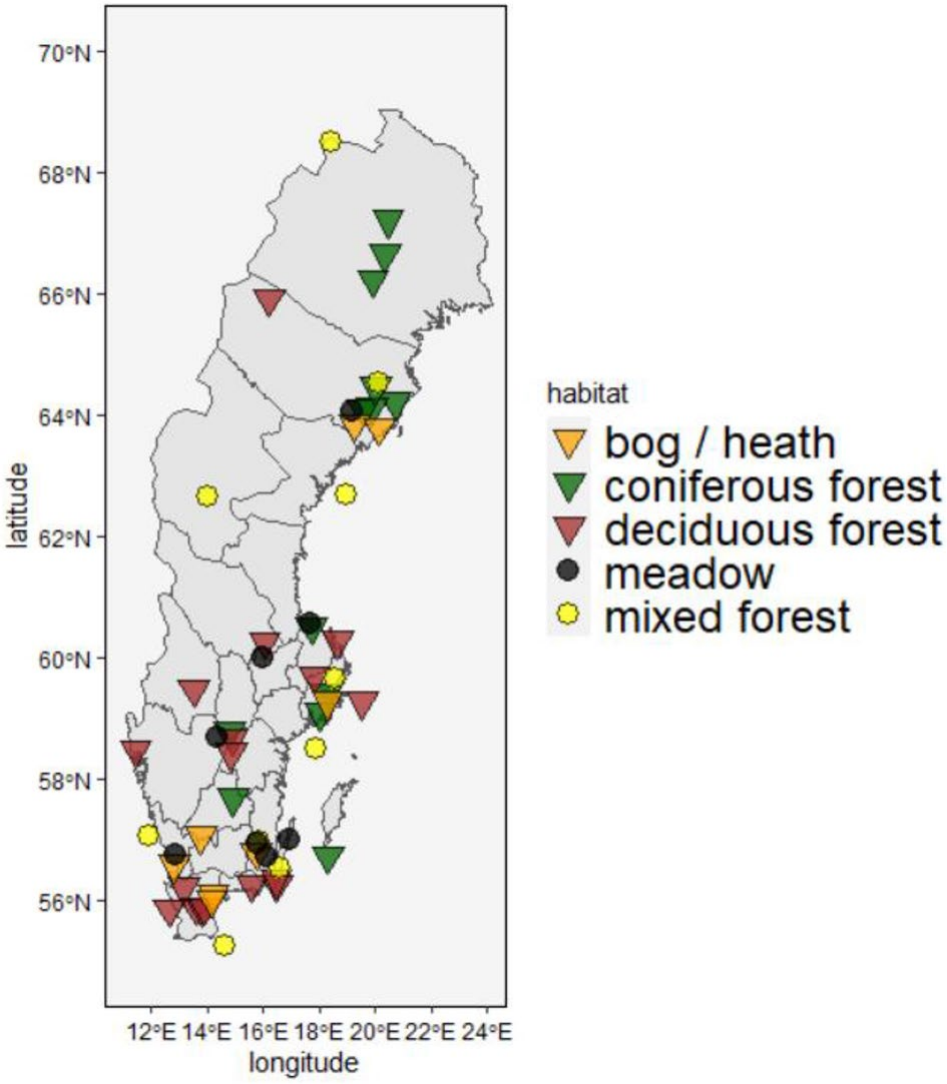
Distribution and habitat of Malaise trap samples in Sweden provided by the Swedish Malaise Trap Project for this study

Oribatid mites from Malaise traps were collected by the ‘Station Linné’, in the framework of ‘The Swedish Malaise Trap Project’ (SMTP, http://www.stationlinne.se/sv/forskning/the-swedish-malaise-trap-project-smtp), covering various habitats and locations across Sweden (Fig. 1). The habitats included three forest types (coniferous, deciduous and mixed), bog/heath sites and meadows. Samples were collected throughout the year and categorized into four seasons: spring (March to May), summer (June to August), autumn (September and October) and winter (November to February). Winter samples had Acari but no oribatid mites and were therefore excluded from further analyses. The numbers of samples differed for seasons (58 collection vials) and habitats (69 collection vials) because traps with sampling dates that did not match the season categories, e.g., taken from May to July, were included in the habitat analyses, but excluded from the season analyses. Samples that did not match the habitat categories of coniferous, deciduous and mixed forests, bog/heath or meadows were excluded from the habitat analyses but included in the season analyses (Table 1). Species that occurred only in one sample were excluded from further analyses.

**Table 1:** Summary of oribatid mite species determined in this study that were collected in Malaise traps by the Swedish Malaise Trap Project (SMTP) from 2003 to 2006, and their occurrence in various habitats and seasons

Species/taxon	Life-style		Habitat					Season		
			Conif	Decid	Mixed	Bog/ heath	Meadows	Spring	Summer	Autumn
**Desmonomata**										
* Camisia biurus* (C.L. Koch)	Meadows, bogs	A	5	–	–	22	–	–	–	27
* Camisia segnis* (Hermann)	Arboricol	A	3	1	–	–	–	–	2	3
* Camisia spinifer* (C.L. Koch)	Soil, arboricol		–	–	–	–	1	–	–	–
* Heminothrus longisetosus* Willmann	Mosses, soil		13	–	–	–	–	–	1	10
* Trhypochthonius cladonicola* (Willmann)	Mosses, lichens, heath	A	–	–	–	13	–	–	13	–
**Brachypylina**										
* Adoristes ovatus* (C.L. Koch)	Soil	S	–	1	–	–	–	–	–	–
* Carabodes labyrinthicus* (Michael)	Mosses, arboricol	A	45	27	3	1	1	–	54	17
* Chamobates pusillus* (Berlese)	Soil, heath	S	–	21	–	–	–	–	11	–
* Cepheus latus* C.L. Koch*	Mosses, soil	A	4	–	–	–	–	–	3	–
* Ceratoppia bipilis* (Hermann)	Soil	S	–	–	1	–	–	1	–	–
* Cymbaeremaeus cymba* (Nicolet)*	Mosses, lichens, arboricol	A	5	7	15	1	1	-	6	6
Damaeidae Berlese	Soil		6	5	1	–	–	–	12	–
* Diapterobates humeralis* (Hermann)	Arboricol	A	13	12	44	46	62	24	148	9
* Eueremaeus valkanovi* (Kunst)*	Mosses, soil, arboricol	A	–	–	2	–	–	–	–	1
* Eupelops acromios* (Hermann)	Soil, arboricol		–	–	–	–	1	1	–	–
* Eupelops strenzkei* (Knülle)*	Meadows	A	–	–	–	2	–	–	2	1
* Galumna alata* (Hermann)*	Mosses, soil, meadow		–	–	–	1	–	–	1	–
* Galumna lanceata* (Oudemans)	Soil		–	331	2	–	–	–	331	2
* Jugatala angulata* (C.L. Koch)*	Arboricol	A	5	1	36	–	–	3	1	–
* Liacarus coracinus* (C.L. Koch)*	Soil	S	–	1	-	–	–	–	1	–
* Odontocepheus elongatus* (Michael)*	Soil	S	–	3	-	–	–	–	3	–
* Oribatella quadricornuta* Michael	Soil, arboricol		18	27	3	–	–	–	41	1
* Oribatula interrupta* (Willmann)*	Mosses, lichens	A	3	–	–	–	–	–	2	1
* Phauloppia lucorum* (C.L. Koch)	Mosses, lichens, arboricol	A	4	21	15	5	–	16	9	–
* Phauloppia nemoralis* (Berlese)	Arboricol		2	2	2	4	–	-	8	–
* Scheloribates ascendens* Weigmann & Wunderle*	Arboricol	A	2	4	6	–	–	–	5	5
* Scheloribates circumcarinatus* Weigmann & Miko*	Soil, meadows, bogs		–	–	–	1	–	–	1	–
* Scheloribates latipes* (C.L. Koch)*	Soil	S	–	–	–	1	–	–	1	–
* Siculobata leontonycha* (Berlese)	Arboricol	A	5	4	–	–	–	4	1	–
* Sphaerozetes piriformes* (Nicolet)*	Mosses, arboricol	A	–	1	–	6	–	–	7	–
* Trichoribates incisellus* (Kramer)*	Meadows	A	–	–	–	–	–	–	6	–
* Trichoribates novus* (Sellnick)*	Meadows	A	–	1	–	–	3	1	2	1
* Xenillus discrepans* Grandjean*	Soil	S	–	6	1	–	–	–	5	2
* Zygoribatula exilis* (Nicolet)	Mosses, arboricol	A	4	–	1	–	–	1	4	–
**Total number of species**			16	19	14	12	6	8	28	14
**Total number of individuals**			137	476	132	103	69	51	681	86

Life-styles were taken from Weigmann (2006), abbreviations A (arboricol) and S (soil) indicate species with clear habitat associations of above- and belowground life-styles; only these species were considered for comparing below- and aboveground-living species in traps. Locations of Malaise traps were categorised into five habitats (*Conif *coniferous forests, *Decid *deciduous forest, *Mixed *mixed forest, Bog/heath, Meadows). All samples were pooled over the years 2003–2006. Asterisks (*) indicate species that were recorded for the first time from Sweden according to Lundquist (1987) and the species list of SMTP (pers. comm.)

Oribatid mites were determined to species level according to Weigmann (2006). Voucher specimens were mounted on cavity slides in Hoyer’s medium and are archived at Station Linné. Finally, life-styles of oribatid mite species were categorized into soil-living species that typically occur on and in the soil, and aboveground-living species that inhabit trees, bark and mosses or lichens (according to Weigmann 2006) (Table 1). We considered mosses and lichens as aboveground habitats, irrespectively if they grow on trees or near the ground, because they differ from the typical detritus-based habitat of soil-living oribatid mites.

### Statistical analysis

Before statistical analysis of differences between oribatid mite communities, densities of all oribatid mite species were log-transformed to improve homogeneity of variances. However, data were still not normally distributed (Shapiro–Wilk-test, p > 0.05), and we therefore used non-parametric multidimensional scaling (NMDS) for investigating the effects of habitat (coniferous forests, deciduous forests, mixed forests, bog/heath, meadows) and season (spring, summer, autumn) on oribatid mite community structure, followed by discriminant function analysis (DFA). NMDS served to reduce the number of variables (species) of the dataset. The quality of the NMDS was indicated by stress values. Stress values indicate how strongly the objects in the compressed matrix differ from the originally calculated distances. By comparing the actual stress values with a theoretical exponential function of stress a meaningful number of dimensions was evaluated. In our case NMDS reduced the number of meaningful dimensions to five for the factor habitat (stress value = 0.0303) and to three for the factor season (stress value = 0.0539). The coordinates of the samples were subsequently used in a DFA with habitat or season as grouping variable. Squared Mahalanobis distances between group centroids and the reliability of the sample classifications were determined.

Subsequently, a detrended correspondance analysis (DCA) was used to analyse (and to present graphically) the response of oribatid mite taxa at the five habitats and the three seasons. The habitats and the seasons were coded as supplementary variables (i.e., they did not contribute to the ordination) and included in the analysis using the passive analysis procedure in CANOCO 5.12 (Jongman et al. 1995; Microcomputer Power, Ithaca, NY, USA, 2012; Lepš and Šmilauer 2020). Total variation accounted for 12.5 and 7.3% for the five habitats and the three seasons, respectively. The data analysis using NMDS and DFA was carried out using Statistica v.13.5 (TIBCO Data Science) and R (R Core Team 2020).

## Results

In total, 962 individuals belonging to 34 species of oribatid mites of the taxa Brachypylina (29 species) and Desmonomata (five species; Table 1) were sampled. These included 331 individuals of *Galumna* cf. *lanceata* from a single sample and twelve individuals of Damaeidae that were determined only to genus level.

Most oribatid mite species occurred in forest habitats (Table 2), i.e., 19 species in deciduous, 18 in coniferous and 13 in mixed forests, and 12 species occurred in bog/heath and nine in meadows. Across seasons, most oribatid mite species occurred in summer (27), eight species occurred in spring, and 14 species in autumn (Table 2).

**Table 2:** Number of oribatid mite species and number of samples containing oribatid mites from Malaise traps collected by the Swedish Malaise Trap Project (SMTP) from 2003 to 2006 in various habitats and seasons

Habitat	No. species	No. samples	Season	No. species	No. samples
Conif	18	18	Spring	8	9
Decid	19	20	Summer	27	39
Mixed	13	12	Autumn	14	10
Bog/heath	12	12	Winter	0	0
Meadows	9	7	Total		58
Total		69			

Locations of Malaise traps were categorised into five habitats (*Conif *coniferous forests, *Decid *deciduous forest, *Mixed *mixed forest, Bog/heath, Meadows)

We assigned life-styles to 31 species; 24 species (77.4%) were characterized as aboveground-living and seven species (22.6%) as soil-living (Table 1). With 534 individuals (93.8% of total) the abundance of aboveground-living species considerably exceeded that of soil-living taxa (35 individuals, 6.1%). *Galumna lanceata* was not included in this comparison because it was very abundant in one sample (n = 331); Damaeidae were also excluded because they were not determined to species level.

### Habitats

Oribatid mite communities differed between the five habitats (DFA using the five dimensions of the NMDS: Wilks’ lambda = 0.47, approx. F_20,199_ = 2.52, p = 0.006). Communities from bog/heath differed from coniferous (squared Mahalanobis Distances (MD) = 3.56; F_5,60_ = 4.8, p < 0.001) and from deciduous forests (MD = 2.98; F_5,60_ = 4.2, p < 0.01). Meadow communities differed from coniferous (MD = 5.3; F_5,60_ = 4.9, p < 0.001) and from deciduous forests (MD = 3.6; F_5,60_ = 3.5, p < 0.008).

**Fig. 2:**
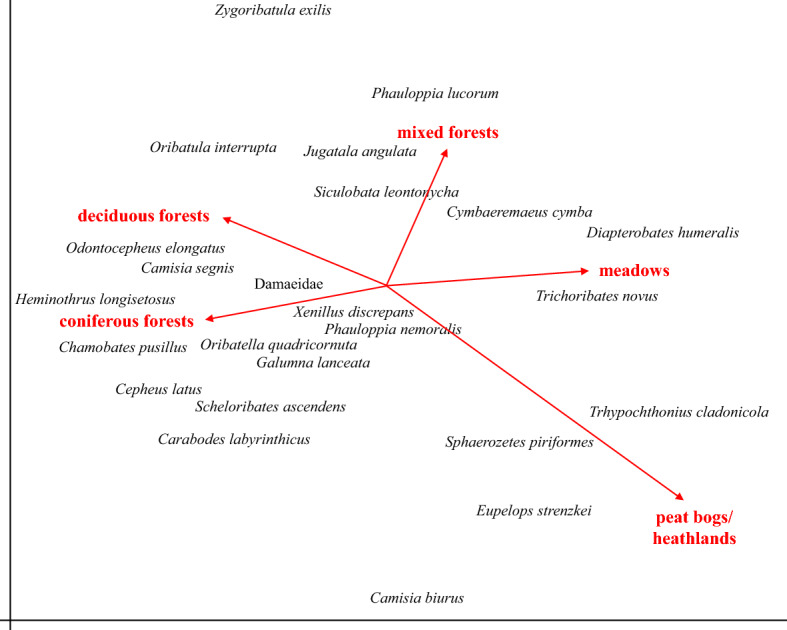
Detrended correspondence analysis (DCA) of oribatid mite species from Malaise traps from five habitats in Sweden (coniferous forests, deciduous forests, mixed forests, meadows and bog/heath). The habitats were included in the ordination as supplementary variables (length of gradient = 6.4; eigenvalues of axis 1 = 0.84 and axis 2 = 0.73)

Detrended correspondence analysis (DCA) clearly separated oribatid mite communities of the five habitats (Fig. 2). Coniferous forests were mainly associated with *Carabodes labyrinthicus, D. humeralis, Heminothrus longisetosus, Cepheus latus* and *Oribatella quadricornuta*. Deciduous forests were mainly associated with *Galumna lanceata, C. labyrinthicus, Chamobates pusillus, D. humeralis, O. quadricornuta* and *Phauloppia lucorum*. The mixed forest was mainly associated with *P. lucorum, Jugatala angulata, Cymbaeremaeus cymba* and *D. humeralis*. The bog/heath habitats were mainly associated with the four species *Camisia biurus*, *Eupelops strenzkei, Sphaerozetes piriformes* and *Trhypochthonius cladonicola*. Only four oribatid mite species occurred regularly at the meadow habitats with *D. humeralis* being the most abundant, whereas *Trichoribates novus, C. cymba* and *C. labyrinthicus* occurred at low densities (Table 1; Fig. 2). The oribatid mite species *D. humeralis* was omnipresent across all habitats.

### Seasons

Oribatid mite communities differed between the three seasons (DFA using the three dimensions of the NMDS: Wilks’ lambda = 0.53, approx. F_10,102_ = 3.73, p = 0.003). Oribatid mite communities from spring differed most from communities in autumn (Squared Mahalanobis Distances (MD) = 7.36; F_5,51_ = 6.5, p < 0.0001), but less from those in summer (MD = 3.11; F_5,51_ = 4.2, p < 0.003). Summer and autumn communities also differed (MD = 1.86; F_5,51_ = 2.7, p < 0.029).

**Fig. 3:**
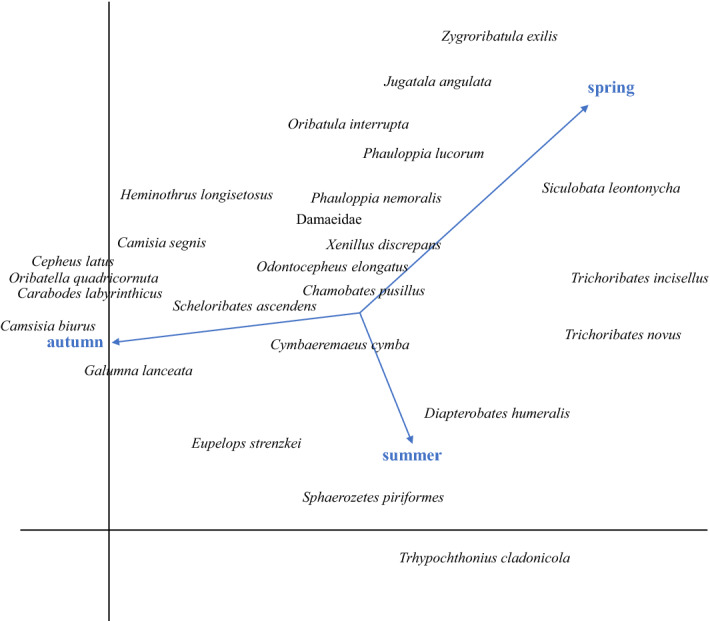
Detrended correspondence analysis (DCA) of oribatid mite species from Malaise trap samples collected in three seasons (spring, summer and autumn). The three seasons were included as supplementary variables (length of gradient = 6.3; eigenvalues of axis 1 = 0.86 and axis 2 = 0.75)

Detrended correspondence analysis (DCA) clearly separated oribatid mite communities of the three seasons (Fig. 3). Spring was mainly associated with *Zygoribatula exilis*, *J. angulata*, *P. lucorum* and *Siculobata leontonycha*; summer was mainly associated with *T. cladonicola, S. piriformes, D. humeralis, E. strenzkei, T. novus;* and autumn was mainly associated with *C. biurus, Camisia segnis, H. longisetosus, C. labyrinthicus, C. latus,* and *Scheloribates ascendens* (Table 1; Fig. 3).

## Discussion

This study investigated the dispersal of oribatid mites in different habitats and seasons based on collections from Malaise traps across Sweden. The design of Malaise traps precludes transportation by wind into the traps, but a combination of passive wind-dispersal onto the traps, followed by active walking into the traps is possible. Malaise traps therefore require wingless animals to enter the trap vials by walking or phoresy, and mainly catch oribatid mite species that have a stronger tendency for walking than others or that actively attach to insects for transport.

Both, oribatid mite species living below and above the ground entered the traps. However, the latter dominated in number of species and abundance indicating that actively moving oribatid mite species or those engaging in phoresy are more abundant in above- than in belowground habitats. Confirming this observation, belowground oribatid mite species have been reported to avoid moving upwards from soil (Proctor et al. 2002; Karasawa et al. 2005; Lindo and Winchester 2006).

Oribatid mite communities in Malaise traps significantly differed among forests, meadow and bog/heath, indicating that active dispersal in oribatid mites varies between habitats. *Camisia biurus, T. cladonicola* and *E. strenzkei* only occurred in bog/heath, *T. novus* only in meadows. *Heminothrus longisetosus, C. latus* and *O. interrupta* occurred only in coniferous forests, while *C. pusillus* and *O. elongatus* were only present in traps from deciduous forests. This supports our hypothesis that dispersal in these oribatid mite species is limited to their local habitat, precluding long-distance dispersal. Dispersal limitation has been suggested as important driver of oribatid mite community composition at the landscape scale (Pequeno et al. 2021) and in fragmented habitats (Åström and Bengtsson 2011), which also agrees with our findings that several species in Malaise traps only occurred in one habitat.

Contrasting the habitat-specific dispersal, four species (*C. cymba, C. labyrinthicus, D. humeralis, P. lucorum*) were common in almost all habitats. *Diapterobates humeralis* and *P. lucorum* are common in forests, but also in Swedish bogs and mires (Tarras-Wahlberg 1961), explaining their omnipresence in Malaise traps across habitats which they may have reached without long-distance dispersal. In a study investigating passive wind-dispersal of oribatid mites in Germany, Lehmitz et al. (2011) found *D. humeralis* and *C. labyrinthicus* to be absent or rare despite their wide distribution in the study area. Both species have been observed to actively walk towards microhabitats of favourable microclimatic conditions (Tarras-Wahlberg 1961; Prinzing et al. 2004; Prinzing 2005), indicating that they also reached Malaise traps by walking. By contrast, *C. cymba* was recorded from sticky traps in the study of Lehmitz et al. (2011), suggesting that this species does disperse over longer distances by wind. However, *C. cymba* likely represents a species complex of genetically deeply divergent but morphologically similar lineages (Schäffer et al. 2019), which suggests limited dispersal ranges within this complex. *Cymbaeremaeus cymba* in our study therefore may also have entered the traps predominanly by walking as this species also lives on the bark of trees (Weigmann et al. 2006, Schäffer et al. 2019), and trees were present in all habitats, albeit less common in meadows and bog/heath habitats than in forests.

Oribatid mite communities in the Malaise traps differed significantly between seasons with spring communities being most different from autumn communities. Further, oribatid mite abundance were highest in summer, lower in spring and autumn and absent in winter. This differed from findings from Malaise traps in the canopies of a Canadian Sitka spruce forest, where species occurred throughout the year (Behan-Pelletier and Winchester 1998). Changes in abundance in our samples also contrasts patterns of soil-living oribatid mites in a German beech forest, where abundance increased in spring and late autumn and declined in summer and winter (Wunderle 1992 and references therein), but also abundances of arboreal oribatid mites in a Japanese cedar plantation, which were rather constant throughout the year (Hijii 1987), similar to soil-living species (Yoshida and Hijii 2005, 2011). However, oribatid mite abundance in canopies of a subtropical rainforest increased from the rainy season in summer to dry periods in autumn and winter (Walter and O’Dowd 1995). Overall, these findings suggest that seasonal fluctuations of oribatid mite abundance above- and below the ground may be habitat specific. An explanation for the strong seasonality of oribatid mite communities in our samples may be that habitats such as trees, mosses and lichens are exposed temporarily to drought, resulting in increased dispersal activity (Prinzing 2005; Lindo and Winchester 2009; Markkula et al. 2019; Wehner et al. 2018). In fact, drought has been identified as important environmental factor affecting oribatid mite abundance and community composition (Lindo and Winchester 2006, 2009; Lindo et al. 2012).

Further, feeding on spatially and temporarily limited resources such as lichens, fungi and mosses (Prinzing 2005; Bailey et al. 2018) may force oribatid mite species to switch diet between seasons, resulting in species-specific dispersal and therefore in different community compositions and abundances across seasons (Lindo and Stevenson 2007). Species feeding on arboricol fungi and mosses such as *S. ascendens* and *Z. exilis* (Bluhm et al. 2015) occurred primarily in summer and autumn. Other oribatid mite species which feed on lichens occurred in spring but not in autumn (*P. lucorum*) or in summer and autumn but not in spring (*C. cymba* and *C. labyrinthicus*) (Maraun et al. 2011; Bluhm et al. 2015). Oribatid mites may feed on different parts and on different functional groups of lichens that also differ in availability or accessibility throughout the year (Lindo and Stevenson 2007), suggesting that seasonal changes in resource availability drives oribatid mite dispersal and affects community composition and abundances in Malaise traps. By contrast, *D. humeralis* was present in traps throughout the year but especially abundant in summer. This species was described as potential pest control agent in Japan that effectively destroys broods of hemlock woolly adelgid aphids by feeding on the woolly filaments that surround the ovisacs (McClure 1995). Possibly, *D. humeralis* feeds on similar resources in northern Europe, only present at certain times in the year, and therefore need to disperse more intensively than other species.

Evidence for phoresy remains equivocal in our data. Occasional findings of *C. cymba* in bark beetle pheromone traps suggests that this species may disperes by phoresy, but this has been questioned (Schäffer et al. 2019). Similarly, *P. lucorum* was found to be phoretic on the bark beetle *Pityokteines curvidens* in Croatia, but this has been assumed to be rare (Pernek et al. 2012). *Diapterobates humeralis* was common in pheromone traps in Finland (Penttinen et al. 2013), and therefore may also engage in phoretic dispersal in our study area*.* The well-known phoretic species *Siculobata (Paraleius) leontonycha* (Ermilov and OConnor 2020; Schäffer and Koblmüller 2020) also occurred in our samples, indicating that phoretic dispersal of oribatid mites did occur. However, the importance of phoretic dispersal in our study is difficult to judge because phoretic associations of oribatid mites with bark beetles and other insects usually is unspecific, varies locally (Knee et al. 2013) and oribatid mites easily fall off from their hosts (Pernek et al. 2012). Therefore, it remains to be investigated which oribatid mite species rely on phoretic dispersal. Correlating the data on oribatid mites from this study with insect data from the same traps would be an interesting first step to narrow down the choice of potential insect hosts for oribatid mite phoresy.

Overall, results of this study indicate that oribatid mites are restricted in their dispersal range to the local habitat they live in. Further, species-specific dispersal likely relies on fluctuating abiotic conditions, such as drought in summer, and feeding on different, temporarily restricted resources. To explain the patterns of seasonal fluctuations among communities it is important to identify the main food resources of different species throughout the year, e.g., by using stable isotope or molecular gut content analyses. In conclusion, results of this study point to the importance of local dispersal within habitats for many oribatid mite species living above the ground, rather than to wide-spread dispersal among habitats, irrespective of the actual mode of dispersal. Presumably, active dispersal via walking or dispersal via phoresy are most important, highlighting the importance of active dispersal for above-ground living oribatid mite species. Notably, 16 species of oribatid mites collected in our study were sampled for the first time in Sweden. This study demonstrates that Malaise traps are a meaningful tool to investigate spatial and temporal patterns of oribatid mite communities.

## Data Availability

Available in the text.
